# Combining Pickering Emulsion Polymerization with Molecular Imprinting to Prepare Polymer Microspheres for Selective Solid-Phase Extraction of Malachite Green

**DOI:** 10.3390/polym9080344

**Published:** 2017-08-06

**Authors:** Weixin Liang, Huawen Hu, Pengran Guo, Yanfang Ma, Peiying Li, Wenrou Zheng, Min Zhang

**Affiliations:** 1 College of Materials Science and Energy Engineering, Foshan University, Foshan 528000, China; lweixin920@126.com (W.L.); huawenhu@126.com (H.H.); lipeiying1108@163.com (P.L.); 13450769881@163.com (W.Z.); 2 Guangdong Provincial Public Laboratory of Analysis and Testing Technology, China National Analytical Center (Guangzhou), Guangzhou 510070, China; prguo@fenxi.com.cn

**Keywords:** pickering emulsion polymerization, molecular imprinting, molecularly imprinted polymer microspheres, specific adsorption, solid-phase extraction column

## Abstract

Malachite green (MG) is currently posing a carcinogenic threat to the safety of human lives; therefore, it is highly desirable to develop an effective method for fast trace detection of MG. Herein, for the first time, this paper presents a systematic study on polymer microspheres, being prepared by combined Pickering emulsion polymerization and molecular imprinting, to detect and purify MG. The microspheres, molecularly imprinted with MG, show enhanced adsorption selectivity to MG, despite a somewhat lowered adsorption capacity, as compared to the counterpart without molecular imprinting. Structural features and adsorption performance of these microspheres are elucidated by different characterizations and kinetic and thermodynamic analyses. The surface of the molecularly imprinted polymer microspheres (M-PMs) exhibits regular pores of uniform pore size distribution, endowing M-PMs with impressive adsorption selectivity to MG. In contrast, the microspheres without molecular imprinting show a larger average particle diameter and an uneven porous surface (with roughness and a large pore size), causing a lower adsorption selectivity to MG despite a higher adsorption capacity. Various adsorption conditions are investigated, such as pH and initial concentration of the solution with MG, for optimizing the adsorption performance of M-PMs in selectively tackling MG. The adsorption kinetics and thermodynamics are deeply discussed and analyzed, so as to provide a full picture of the adsorption behaviors of the polymer microspheres with and without the molecular imprinting. Significantly, M-PMs show promising solid-phase extraction column applications for recovering MG in a continuous extraction manner.

## 1. Introduction

Malachite green (MG) is an *N*-methylated diaminotriphenylmethane dye widely used in the fish and dye industries due to its availability, low cost, and sterilization efficacy in killing bacteria and parasites [1,2]. On the other hand, a large number of studies have demonstrated that MG exhibits a high toxicity to organisms [1,2,3,4,5,6,7,8,9,10], and can pose a carcinogenic and mutagenic threat to humans through bio-enrichment [1,5,7]. Even worse, the recalcitrant nature of MG indicates that it can be enriched persistently in the organism body, causing a long-term toxicity [6]. Despite being prohibited in the People’s Republic of China in 2002, the pesticide MG is still widely used [11,12]. Therefore, it is highly significant and important to explore an effective method for fast trace detection of MG in water media. In this context, a number of approaches have been reported, including high-performance liquid chromatography-fluorescence detection and ultra-performance liquid chromatography-tandem mass spectrometry [11,12]. However, the pretreatment of these methods is rather complicated due to the involved procedures of breaking the fish sample, extraction with acetonitrile, concentration by rotary evaporation, purification with solid phase extraction column, and final isolation and detection in the liquid chromatograph and mass spectroscope, respectively. Currently, cation exchange columns are commonly used for solid phase extraction due to a lack of specific adsorption interactions with MG and hence causing a complex pretreatment process. Alternatively, using molecularly imprinted materials for solid phase extraction can largely simplify the pretreatment process, which is thus highly desirable for fast trace detection of MG [13,14].

Bulk polymerization is commonly used for producing molecularly imprinted materials, but they exhibit a bulk form, thus needing ball-milling treatment to obtain the fine particles [11,12]. Generally, the obtained particles show irregular shapes, resulting in tight packing and hence being unfavorable for fast adsorption. In addition, precipitation polymerization is also investigated to produce molecularly imprinted materials, but a large amount of environmentally unamiable organic solvents are involved [15]. Alternatively, Pickering emulsion polymerization involves the use of solid nanoparticles as an interfacial adsorbent, which can lead to the generation of stable polymer microspheres with the reduced amount of or even without any organic emulsifiers being commonly used in traditional emulsion polymerization processes [16,17,18,19], leaving a smaller environmental footprint [17].

Recently, a number of molecularly imprinted materials have been explored [20,21,22], e.g., polymer microspheres prepared using modified SiO_2_-stabilzied atenolol, metoprolol, timolol, pindolol, and 1-naphthol as the template [23], microspheres synthesized with magnetic particles and lignin-stabilized cyhalothrin as the template [24,25], microspheres obtained by employing chitosan-stabilized erythromycin as the template [26], and microspheres produced with cellulose nanocrystals-stabilized bifenthrin as the template [27]. These reported molecularly imprinted materials are commonly used for sustained drug release applications. To our knowledge, using Pickering emulsion polymerization as the method and MG as the template for preparation of polymer microspheres (used for detection and selective purification of MG) has not been reported so far, and the use of such polymer microspheres for solid phase extraction applications has also not been found through the literature review [28,29,30,31,32,33,34,35,36,37,38,39,40,41,42,43,44,45,46,47].

By virtue of molecular imprinting, the as-generated molecularly imprinted polymer microspheres (M-PMs), with a designed shape of space, can specifically recognize and enrich an organic pollutant, in this case MG. The procedures of molecular imprinting involve (i) assembly of functional monomers in a solvent containing MG template molecules, (ii) formation of a polymer with a composite network and specific space structure through Pickering emulsion polymerization of the monomers, and (iii) removal of the target molecules from the formed polymer by solvent extraction, leaving a hole structure with a space shape and chemical properties complementary to the MG molecules [47]. Consequently, such a hole structure exhibits specific recognition capability for MG, facilitating the specific binding with the MG molecules. This memory of M-PMs toward the shape and functionalities of the MG molecules makes it promising for detecting and selectively removing MG from a contaminated source area [48].

This study presents selective adsorption and purification of MG using M-PMs with hydrophilicity, which is superior to hydrophobic M-PMs (as prepared using organic solvents) and suitable for processing water-borne MG. Importantly, the preparation and applications of hydrophilic M-PMs are the focus of the current scientific and engineering research [47]. The structure and adsorption properties (toward MG) of the designed hydrophilic M-PMs are systematically investigated. The design of such M-PMs specifically targeting to MG can open up exciting opportunities for monitoring and selectively purifying many other kinds of hazardous molecules that pose a serious threat to health. The present M-PMs are also expected to show a wide range of other promising applications such as detection and processing of aqueous solutions containing various dye molecules, veterinary drugs, pesticide residues, proteins, amino acids, or/and antibiotics. Furthermore, M-PMs can also be used as promising drug carriers for biomedical applications [47].

## 2. Experimental

### 2.1. Materials

Fumed silica (Aerosil 200) of 99% purity and with an average size of 12 nm was supplied by Evonik Degussa (Evonik Industries AG Inorganic Materials, Hanau, Germany). 2,2′-azoisobutyronitrile (AIBN, 99% purity) was obtained from Tianjin Fucheng Chemical Reagent Factory (Tianjin, China). Triton-100 of 99% purity was purchased from Sangon Biological Engineering Co., Ltd. (Shanghai, China). MG, rosolic acid (RA), and basic yellow 1 (BY) were AR grades and obtained from New Fine Chemicals Development Center (Tianjin, China). Deuterated MG (99.8% purity) and leuco-malachite green (Leuco-MG) of 99.0% purity were purchased from WiTEGA (Dr. Ehrenstopfer GmbH, Augsburg, Germany). Ethylene glycol dimethyl acrylate (EGDMA) of 98% purity was obtained from Aladdin Reagent (Shanghai, China). Sodium hydroxide (NaOH), toluene, hydrofluoric acid (HF), hydrochloride acid (HCl), glacial acetic acid, and anhydrous ammonium acetate were AR grades and supplied by Tianjin Damao Chemical Reagent Factory (Tianjin, China). Methanol and acetonitrile of chromatographic purity were purchased from Tianjin Siyou Fine Chemicals Co., Ltd. (Tianjin, China).

### 2.2. Preparation of M-PMs Specifically Targeted to MG

**Figure 1:**
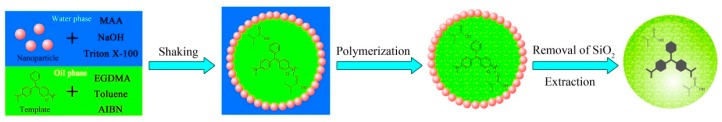
Schematic illustration of the synthesis of M-PMs by Pickering emulsion polymerization and molecular imprinting.

The schematic illustration of the preparation of hydrophilic M-PMs by Pickering emulsion polymerization is shown in Figure 1 and Figure S1 of Electronic Supporting Information (ESI). The preparation of the M-PMs mainly includes the following steps: (i) preparation of the Pickering emulsion, (ii) free-radical polymerization, and (iii) solvent extraction. Specifically, the water phase used for the emulsion preparation mainly consisted of methacrylic acid (MAA, 0.27 mL), NaOH (3 M, 0.5 mL), Triton X-100 (0.3%, 6 mL), and nanosilica (20 mg). Sonication treatment was used to uniformly disperse SiO_2_ into water. On the other hand, the oil phase was composed of ethylene glycol dimethyl acrylate (EGDMA, 1.728 mL), the template molecules (MG), toluene (0.20 mL) as the pore forming agent, and AIBN as the initiator. To homogenize the mixture in the oil phase, sonication treatment for 10 min was performed. The water and oil phases were then mixed together by intense agitation, leading to the formation of the Pickering emulsion. The free radical polymerization of the monomer MAA in the Pickering emulsion was conducted in a water bath at 70 °C for 16 h. The free-radical polymerization and crosslinking reactions were triggered by AIBN (see the reactions shown in Figure S1 of Supplementary Materials (SM)). The sinking microspheres were cleaned by dipping into a HF solution (30%) for 12 h to remove the silica particles on the surface. The final solvent extraction of the template molecules was achieved using a Soxhlet extractor, with a methanol solution containing 10% acetic acid as the extraction solvent, leading to the generation of M-PMs. The Soxhlet extraction was conducted for 48 h, being sufficiently long to ensure that the MG could not be detected by ESI-MS. A series of M-PMs were prepared using different formulations with various addition ratios of raw materials. Detailed information of the synthesis can be referred in Table 1. Note that Leuco-MG was also used as the template for synthesis of the M-PMs (samples 5 and 6), in addition to MG; this is by considering that Leuco-MG is a structural derivative of MG (they have a similar chemical structure, as shown in Figure S2 of SM); under reductive conditions, MG would readily be transformed to Leuco-MG, while the reversed transformation of Leuco-MG to MG could take place under oxidative conditions. Therefore, apart from MG, it is important and significant to investigate Leuco-MG as the template to synthesize M-PMs and investigate their adsorption performance. Nevertheless, we also found that, when adopted as the template, Leuco-MG could be oxidized to MG during the experiment, causing the resultant polymer microspheres to have inferior uniformity. As a result, sample 3 synthesized by using MG as the template was adopted as the specimen for all the following characterizations and analyses; it is designated as M-PMs for the following descriptions and explanation. For comparison, sample 1, designated as PMs, is used for all the following characterization and analyses, i.e., polymer microspheres prepared without involving the template molecules (MG or Leuco-MG) according to the above procedures except without the template. 

### 2.3. Characterizations

Scanning electron microscopy (SEM) and energy dispersive X-ray (EDX) spectrum of the samples were conducted on a HITACHI S-4800 Scanning Electron Microscope equipped with an EDX spectrometer (Quantax 70, Bruker Nano GmbH, Berlin, Germany). The conditions adopted for the mass spectrometric measurements were given as follows: Atomization gas of 40 psi, drying gas flow of 10 L/min, drying gas temperature of 350 °C, and scanning range of *m*/*z* 100–*m*/*z* 500. The solid extraction device (DG-12B) was East Health Sci. & Tech. Co., Ltd. (East Health Sci. & Tech. Co., Ltd., Tianjin, China). The Brunauer–Emmett–Teller (BET) surface areas of the prepared M-PMs and PMs were analyzed by nitrogen adsorption in a Micromeritics ASAP 2020 nitrogen adsorption apparatus (Norcross, GA, USA); the samples were pre-degassed at 150 °C before the nitrogen adsorption measurements. The corresponding pore size distribution was determined by analyzing the desorption branch using the Barrett–Joyner–Halenda (BJH) method.

### 2.4. Selective Adsorption of MG by M-PMs

To measure the adsorption selectivity of the as-synthesized M-PMs toward MG, a solution containing MG, and two other dyes, rosolic acid (RA) and basic yellow 1 (BY), was investigated. RA has a structure similar to that of MG, while BY presents a different structure from that of MG (Figure S3 of SM). The molecular structures of MG, RA, BY, and Leuco-MG are presented in Figures S2 and S3 of SM. The mass spectrum of the mixed solution containing MG, RA, and BY is also given in Figure S4 of SM. The standard curve of MG is presented in Figure S5 of SM, showing a good linearity with the linear coefficient *R*^2^ as high as 0.999. Specifically, a given amount of M-PMs (0.01 g) was placed in a beaker (50 mL) with a mixed dye solution (0.03 mol/L), and the adsorption interactions were carried out in a thermostatic water bath oscillator for 3 h. The selective adsorption performance of M-PMs was evaluated using distribution coefficient (*K*_d_), selectivity coefficient (*K*) and relative selectivity coefficient (*K*_0_). The following Equations (1)–(3) are adopted to calculate these coefficients.

*K*_d_ = *q_e_*/*C_e_* (1)

*k’* = *K*_d(MG)_/*K*_d(x)_ (2)

*K*_0_ = *k’*_M_/*k’*_N_ (3)

where *K*_d_, *q_e_*, and *C_e_* represent distribution coefficient, equilibrium absorption capacity, and equilibrium mass concentration, respectively. *K* and *K*_d(MG)_ are selectivity and distribution coefficients for MG, respectively. *K*_0_, *K*_M_, and *K*_N_ represent relative selectivity coefficient, selectivity coefficient for M-PMs, and selectivity coefficient for PMs, respectively.

Deuterated MG was used as an interior label, and MG, RA, and BY worked as reference materials. Based on the ionic strength ratio of the reference material to the interior label, correction factor *f* was calculated according to Equation (4), which was subsequently used to estimate the concentration of each component based on Equation (5). The amount of each component adsorbed by M-PMs was finally obtained.

(4) $$f=\frac{A_{s}/C_{s}}{A_{r}/C_{r}}$$

(5) $$C_{i}=\frac{A_{i}}{A_{s}/C_{s}}\times f$$

where *A_s_*, *A_r_*, and *A_i_* represent the ionic strengths of the interior label, reference material, and specimen, respectively, and *C_s_*, *C_r_*, and *C_i_* are the concentrations of the interior label, reference material and specimen, respectively. For comparison, PMs were also measured in a parallel fashion, so as to clarify the molecular printing function for achieving selective adsorption of MG.

### 2.5. Molecularly Imprinted Solid-Phase Extraction (MISPE) Column Test

Used as filler, M-PMs were placed in an empty MISPE column. The impact of the amount of M-PMs on the collecting efficiency for MG and Leuco-MG was investigated. Deuterated MG was used as an interior label for calculation of recovering percentage according to the following Equation (6).

(6) $$P=\frac{C_{2}\times V_{1}}{V_{0}\times C_{0}}$$

where *V*_1_, *C*_0_, *V*_2_, and *C*_2_ represent labeling volume, labeling concentration, diluted solution volume, and real sample concentration as measured, respectively.

### 2.6. Static Adsorption of MG

To investigate the impact of the concentration, and pH value, etc. of the initial solution on the adsorption interactions between MG and M-PMs, MG solutions with different pHs (5.0, 6.0, 7.0, and 8.0) and concentrations (10, 20, 30, 40, 50, and 60 mg/L) were examined. The color depth can be a good indication of the concentration of MG. During adsorption, the MG solution was monitored by UV/Vis spectrophotometry to obtain the absorbance value. The corresponding MG concentration *C_e_* was then estimated using the standard curve of MG shown in Figure S5 of SM. Based on the initial concentration *C*_0_ and the concentration *C_t_* of residual MG after the adsorption, the adsorption amount *q_e_* can be calculated using the following Equation (7).

(7) $$q_{e}=\frac{C_{0}-C_{t}}{m}\times V$$

where *q_e_*, *C*_0_, *C_t_*, *m*, and *V* are the equilibrium adsorption amount (mg/g), initial MG concentration (mg/L), the concentration of MG after the adsorption for time *t* (mg/L) the mass of the adsorbent (g), and the volume of the solution (L), respectively.

### 2.7. Adsorption Kinetics

The adsorption kinetics refer to the investigation of the adsorption rate and hence are also called reaction kinetics. The kinetic investigation includes the study of the influence of different experimental conditions such as temperature, MG concentration, and adsorption time on the adsorption mechanism, which can feature a chemical reaction. The most commonly-used models are first- and second-order kinetic models. The first-order reaction indicates that the reaction rate is dependent upon the concentration of one reactant, while the second-order refers to the reaction rate being in direct proportion to the product of the concentrations of the two reactants. The pseudo-first-order and pseudo-second-order kinetic equations are given below.

The pseudo-first-order kinetic equation:

ln(*q_e_* − *q_t_*) = ln*q_e_* − *tk*_1_ (8)

The pseudo-second-order kinetic equation:

(9) $$\frac{t}{q_{t}}=\frac{1}{k_{2}q_{e}{}^{2}}+\frac{1}{q_{e}}t$$

where *k*_1_, *k*_2_, *q_e_*, and *q_t_* represent pseudo-first-order adsorption rate constant (1/min); pseudo-second-order adsorption rate constant (1/min), equilibrium adsorption amount (mg/g), and the adsorption amount at time *t* (mg/g).

### 2.8. Isothermal Adsorption

(a) Langmuir adsorption isotherm

The Langmuir equation is most-widely used as the adsorption model for explaining the adsorption behaviors of various adsorbents, which can also be applied to surface kinetics and thermodynamics. The Langmuir isothermal adsorption equation is presented as follows Equation (10).

(10) $$q_{e}=\frac{k_{l}C_{e}}{1+q_{m}C_{e}}$$

which can also be presented in a linear form:

(11) $$\frac{1}{q_{e}}=\frac{1}{q_{m}}+\frac{1}{k_{l}C_{e}q_{m}}$$

where *q_e_*, *q*_m_, *C_e_*, and *k*_1_ represent equilibrium adsorption amount (mg/g), saturated mono-layer adsorption amount (mg/g), equilibrium mass concentration (mg/L), and adsorption equilibrium constant (L/mg), respectively.

(b) Freundlich adsorption isotherm

The Freundlich isothermal adsorption equation is presented as the following Equation (12).

(12) $$q_{e}=kC_{e}{}^{1/n}$$

which can also be presented in a linear form:

(13) $$\ln(q_{e})=\ln k+\frac{1}{n}\ln C_{e}$$

where *q_e_*, *C_e_*, *k*, and *n* are equilibrium adsorption amount (mg/g), equilibrium mass concentration (mg/L), adsorption equilibrium constant (L/mg), and another constant, respectively.

### 2.9. Adsorption Thermodynamics

Thermodynamics refer to the science that describes the relationship between temperature and reactions. Thermodynamic functions Δ*G*°, Δ*H*°, and Δ*S*° can be turned to clarify the transfer and change of thermal energy, facilitating the investigation of adsorption mechanism. The values of Gibbs free energy change Δ*G*°, and enthalpy change Δ*H*° can be used to judge whether the adsorption reaction is a spontaneous and exothermic process, respectively. In a solid-liquid system, the adsorbate molecules, as dispersed in the solution, adhere to the solid surface, lowering the chaos of a system and hence reducing entropy. Consequently, Δ*S*° is below 0. The thermodynamic functions can be calculated according to the following equations.

(14) $$K0=\frac{q_{e}}{C_{e}}$$

(15) $${\Delta G}^{{}^{\circ}}={-\mathrm{RT}\;\mathrm{lnK}}_{0}$$

(16) $${\mathrm{RT}\;\mathrm{lnK}}_{0}=T\Delta S{}^{\circ}-\Delta H{}^{\circ}$$

Equation (16) can also be presented in a linear form:

(17) $${\mathrm{lnK}}_{0}=-\frac{\Delta H{}^{\circ}}{R}\frac{1}{T}+\frac{\Delta S{}^{\circ}}{R}$$

where *q_e_*, *K*_0_, and *R* represent quilibrium adsorption amount (mg/g), thermodynamic equilibrium constant, and gas constant (8.314 J/mol·K).

After adsorption equilibrium, the values of *C_e_*, *q_e_*, and *K*_0_ can be calculated. Using ln*K*_0_ as the *y*-coordinate and 1/*T* as the *x*-coordinate, the regression equation can be depicted, and then the value of Δ*S*° can be obtained by means of the *y*-intercept $\frac{\Delta S{}^{\circ}}{R}$, and the same to Δ*H*° through the *y*-intercept $-\frac{\Delta H{}^{\circ}}{R}$.

## 3. Results and Discussion

### 3.1. Structural Characterizations and Analysis

Figure 1 and Figure S1 of SM illustrate the synthesis of M-PMs by Pickering emulsion polymerization. Free-radical polymerization of MAA and crosslinking reactions between MAA and EGDMA can be triggered by the initiator AIBN, leading to the generation of the crosslinking network structure. Ion pairs can be formed between the carboxylate groups of MAA and cationic ions on MG, resulting in effective adsorption of MG. The network structure of M-PMs can immobilize the adsorbed MG at the hole position, forming a molecular imprinting structure on the polymer surface. It was found that the absence of the MG template molecules led to the formation of relatively larger emulsion particles, while smaller emulsion particles could be generated, with an improved stability, in the presence of the template molecules. Such improved emulsion stability most likely results from the MG molecules (as a cationic dye) that can serve as a cationic surfactant and hence are able to stabilize the emulsion. As a consequence, the more uniform surface of the resulting M-PMs, with a smaller average size, can be produced. We also noted that a fine and uniform emulsion could be formed for any system with template molecules either in the water or oil phase. The presence of a little amount of styrene resulted in the formation of floccules, which was unfavorable for the stability of the emulsion.

**Figure 2:**
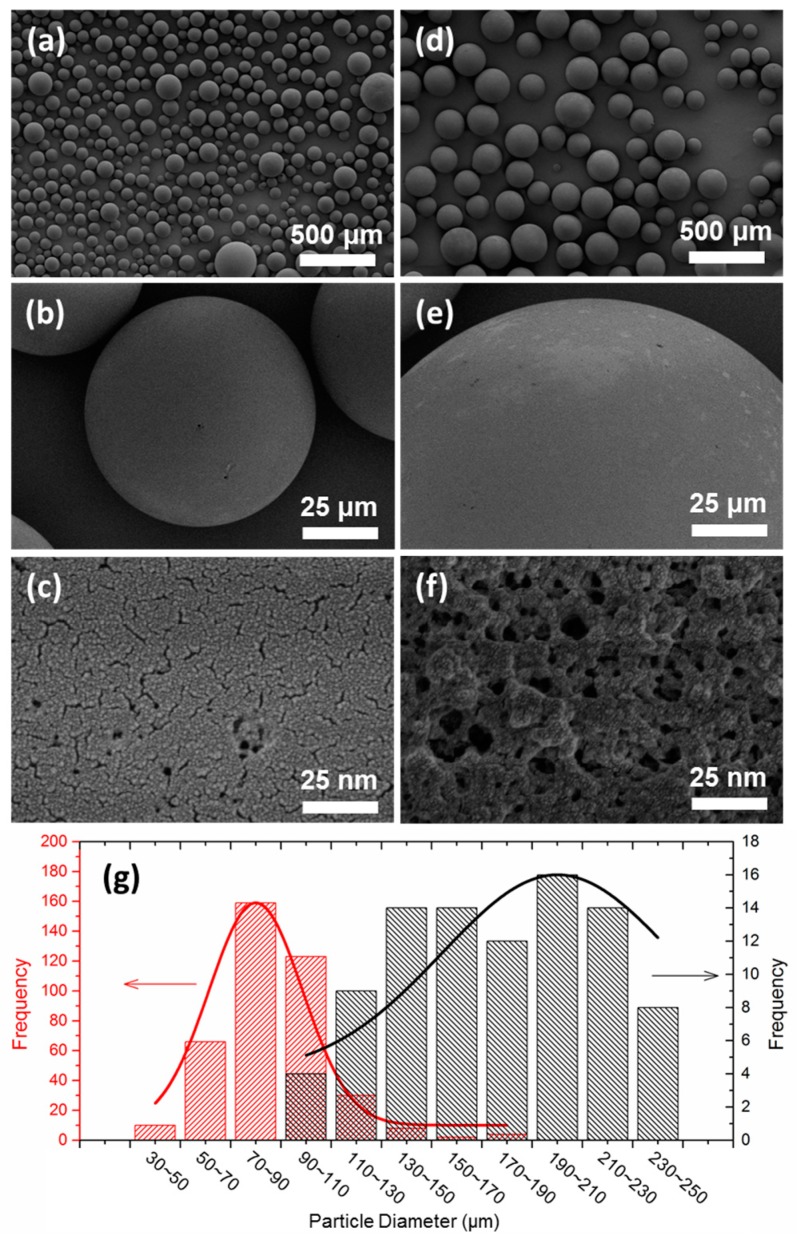
(**a**–**f**) SEM images of M-PMs (**a**–**c**) and PMs (**d**–**f**) at different magnification scales, and (**g**) the particle diameter distribution of M-PMs (red histogram and line) and PMs (black histogram and line).

**Figure 3:**
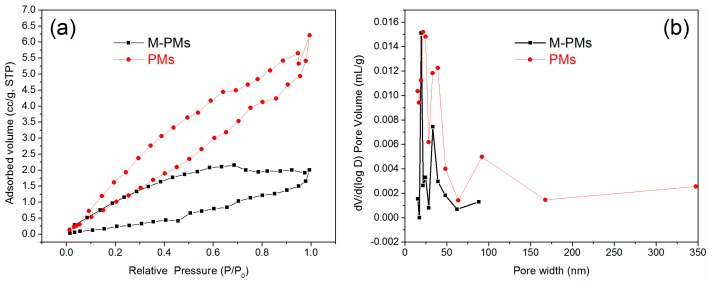
(**a**,**b**) Nitrogen adsorption-desorption isotherms (**a**) and pore size distribution (**b**) of M-PMs and PMs. The corresponding pore size distribution was determined by analyzing the desorption branch using the Barrett–Joyner–Halenda BJH method.

SEM images shown in Figure 2a–f demonstrate that the addition of the template molecules can lead to the reduction of the average particle diameter of M-PMs to 80–90 μm that is almost two times smaller than that of PMs (180–190 μm), especially discernable in particle size distribution histograms together with their fitted lines (Figure 1g). The number of the microspheres counted in each case is given in Table S1 (SM). The polymer microspheres, as synthesized by Pickering emulsion polymerization, present an impressive uniformity, with a regular sphere shape, revealing uniform adsorption sites (Figure 2a–c and Figure 2d–f for M-PMs and PMs, respectively). This is superior to the M-PMs prepared by traditional bulk polymerization that needs ball milling treatment, leading to irregular shapes and destructed adsorption sites [49]. It is of interest to note that the surface of M-PMs show regular porous structure, with uniform pore size distribution (Figure 2c). In contrast, the surface of PMs, as prepared without involving template molecules, exhibits roughness, with pores of irregular shapes and non-uniform size distribution (Figure 2f). The regular pores with uniform size of M-PMs most likely imply a high specificity to adsorbing MG. The N_2_ adsorption-desorption isotherms and pore size distribution patterns for M-PMs and PMs (Figure 3a,b) further demonstrate that M-PMs show a somewhat lower specific surface area and smaller average pore diameter (20–50 nm) as compared to PMs. Such smaller pores most likely reveal the better engineered physicochemical structure of M-PMs by means of molecular imprinting for specific adsorption of MG.

**Figure 4:**
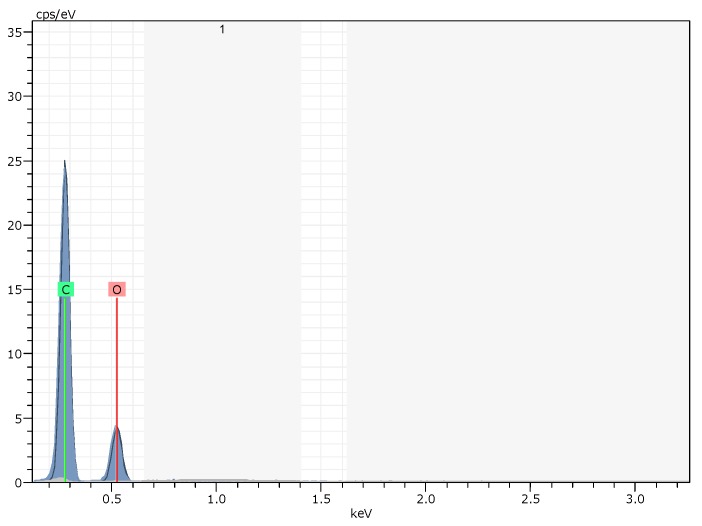
Energy dispersive X-ray (EDX) spectrum of the prepared M-PMs.

The elemental composition of M-PMs can be unraveled by EDS measurements (Figure 4), and only elements C and O can be probed, with the atomic percentages estimated to be 74.62% and 24.38% respectively. Before polymerization, the silica forms an outer layer, while the emulsion containing the monomer MAA occupies the internal layer. The free-radical polymerization leads to the formation of M-PMs with silica as the outer shell that can be etched away by HF (Figure 1). As confirmed by the EDS spectrum (Figure 4), the silica shell can be removed completely by HF, leaving M-PMs with only elements C and O as the composition.

### 3.2. Adsorption Measurements and Analysis

**Figure 5:**
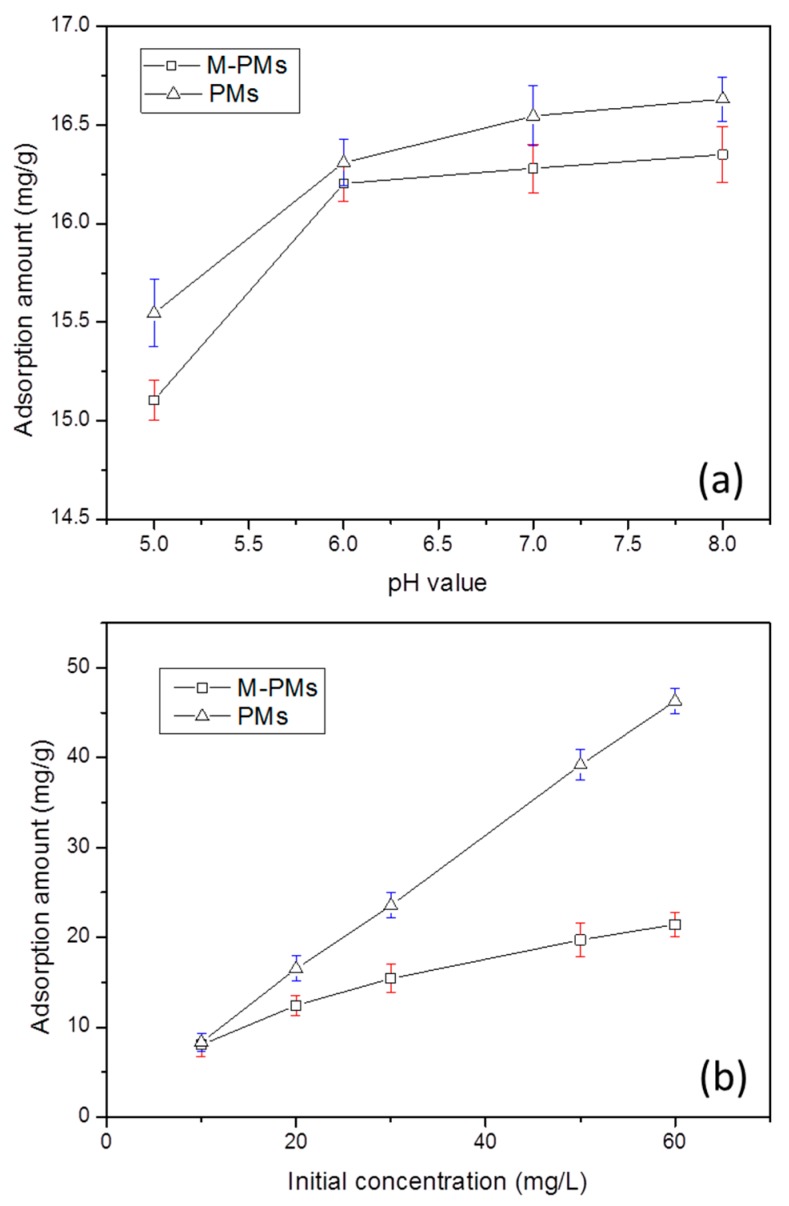
Study of the impacts of pH (**a**) and initial MG concentration (**b**) conditions on the adsorption amount of MG using M-PMs and PMs as adsorbents; the error bars indicate the standard deviation of three independent measurements. The test conditions for (**a**): at 35 °C, initial MG concentration = 20 mg/L, dosage of microspheres = 0.03 g, and volume of the MG solution = 25 mL. The test conditions for (**b**): at 35 °C, pH 7, dosage of microspheres = 0.03 g, and volume of the MG solution = 25 mL.

The pH of the solution has a similar impact on the adsorption performance of M-PMs and PMs, as shown in Figure 5a. A smaller pH value corresponds to a lower adsorption capacity. In the pH range of 5.0–8.0, the adsorption capacities of M-PMs and PMs toward MG are largely increased when changing the solution from acidic to alkaline. At lower pHs, most of the MAA units of M-PMs (pKa 5.85, as presented in Figure S6 of SM) are protonated, and hence the ionic interactions between acid groups of MMA units and cationic MG molecules are largely weakened. When the pH increases, the acid groups of M-PMs would be deprotonated, starting the ionic interactions with the MG molecules, along with the raised adsorption amount of MG. Considering that a high pH could lead to the structural destruction of MG, forming a white flocculent precipitate, the optimal pH condition for the adsorption of MG is found to be in the range of 6.0–7.0, which is just corresponding to the weakly acidic solution containing MG. The optimal pH condition would be adopted for the following adsorption experiment. Figure 5b presents the investigation of the impact of the initial MG concentration on the adsorption performance of M-PMs and PMs. In the concentration range of 10 to 60 mg/L, increasing the initial concentration can facilitate the adsorption for MG. It can be noted that the adsorption capacity of PMs is higher than that of M-PMs, which can be due to the more rough and porous surface of PMs with the higher specific surface area, 5.012 m^2^/g (almost three times higher than 1.808 m^2^/g of M-PMs, as revealed in Figure 3) and larger average pore size (Figure 2 and Figure 3). A stronger micro-pore and-channel effect can thus exist. Therefore, PMs show strong non-specific adsorption interactions with different kinds of substrates as investigated. Nevertheless, this study aimed to develop an adsorbent that exhibits specific adsorption for MG from the mixed adsorbates, and hence M-PMs are more desirable in this case.

**Figure 6:**
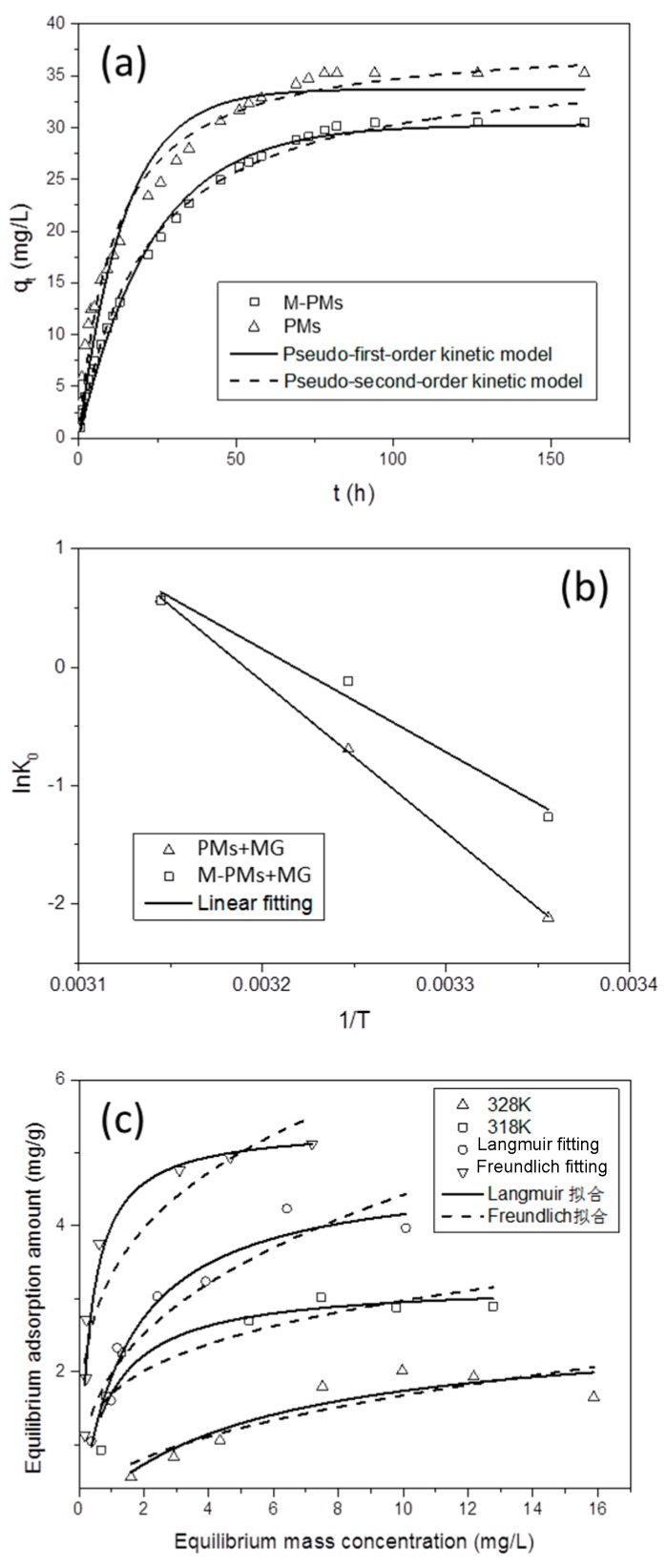
(**a**) Adsorption kinetics of M-PMs and PMs for MG (test conditions: at 35 °C, pH 7, initial MG concentration = 10 mg/L, dosage of polymer microspheres = 0.05 g, and volume of the MG solution = 250 mL). (**b**) Plot of ln*K*_0_ as a function of 1/*T* (test conditions: pH 7, initial MG concentration = 10 mg/L, dosage of polymer microspheres = 0.01 g, and volume of the MG solution = 10 mL). (**c**) Isothermal adsorption patterns for the adsorption of MG by M-PMs, along with the Langmuir and Freundlich fittings (test conditions: pH 7, initial MG concentration = 3 mg/L, dosage of polymer microspheres = 0.01 g, and volume of the MG solution = 4 mL).

The adsorption kinetic characteristics of the prepared M-PMs and PMs for MG are also investigated, as shown in Figure 6a. There are two steps in the adsorption process of M-PMs and PMs toward MG, that is, fast and slow adsorptions within and beyond 60 h respectively. After the two steps, the adsorption amount become saturated and gradually turns out to be stable. It has been reported that four procedures are involved in the adsorption [50]: (i) adsorbate molecules are first diffused over the solution and reach the surface of the adsorbent, (ii) thereafter adhering to the surface of the adsorbent, (iii) are then diffuse into the adsorbent through pores and channels, and (iv) finally adhere to the inner surface of the adsorbent. The procedures (i) and (ii) mainly affect the first step of fast adsorption, since the MG molecules can be quickly adsorbed onto the surface of M-PMs and PMs through diffusion. After the initial adsorption for tens of hours, the procedures (iii) and (iv) take effect by means of slow surface mass transfer of the adsorbate molecules into the inner side of the M-PMs and PMs, followed by adsorption onto the inner surface. Through data fitting (Figure 6a and Table 2), the adsorption of MG by M-PMs satisfies both the pseudo-first-order and pseudo-second-order kinetic models, with both the linear coefficient R^2^ larger than 0.99. In contrast, as for PMs, the adsorption for MG exhibits the linear coefficient R^2^ smaller than 0.99 for both the fitted kinetic models. The pseudo-first-order kinetic model assumes that the diffusion procedure affects the adsorption process, and the mass transfer is the rate-limited step for the adsorption. The adsorption by M-PMs, which can be well-fitted to the pseudo-first-order kinetic model, is most likely due to a compact hydration shell as formed on the surface of M-PMs, thereby impeding the MG molecules from reaching the surface of M-PMs. The pseudo-second-order kinetic model presumes that the electron sharing or transfer takes place between the adsorbent and adsorbate, with the adsorption rate determined by the chemical adsorption. The better-fitted adsorption by M-PMs to the pseudo-second-order kinetic model, as compared to that by PMs, implies the chemical adsorption as the main adsorption interactions between M-PMs and MG. In contrast, both chemical and physical adsorption interactions play a significant role in the PMs-based adsorption for MG. These results can also explain the higher adsorption capacity of PMs than that of M-PMs. On the other hand, it is well known that chemical adsorption exhibits a higher specificity as compared to physical adsorption, thus revealing a higher specificity of M-PMs for the adsorption of MG relative to PMs, despite a lower adsorption capacity.

In the solid-liquid system, the adsorbate molecules being initially dispersed in the solution become gathered at the solid surface, thereby lowering the chaotic degree; this thus reveals that the adsorption reaction is a typical process with a decrease of entropy, namely ΔS° < 0. For the adsorption of MG onto M-PMs and PMs, Δ*H*° is positive (see Figure 6b and Table 3), indicating an endothermic adsorption process. Raising the temperature facilitates the adsorption of MG onto both M-PMs and PMs. This is not consistent with the thermodynamic theory, likely because chemical interactions play a dominant role in the adsorption, and hence a higher temperature can enable an increased adsorption rate. This thus negates the actual exothermic process of the adsorption of MG onto the surface of the adsorbents.

The overall entropy change ΔS° is found to be larger than 0 (Figure 6b and Table 3), confirming that the system is evolved from order to disorder. As aforementioned, the adsorption is a process with a decrease of entropy. Contrarily, the present increased entropy most likely indicates the formation of a water layer before the adsorption of MG molecules onto M-PMs and PMs. The adsorption of MG molecules is thus accompanied by desorption of water molecules. Owing to the smaller molecular weight of water molecules as compared to MG molecules, the number of the desorbed water molecules is much bigger than that of the adsorbed MG molecules, enhancing the disorder of the system. As a consequence, the overall entropy change is positive from the macroscopic viewpoint. This can also explain the overall endothermic process of the adsorption, since desorption of water molecules is an endothermic process, with the energy demand higher than that generated during the exothermic adsorption of MG. Moreover, the change of Gibbs free energy can also reflect these findings well. At 300 and 310 K, ΔG° is positive (Δ*G*° > 0), while Δ*G*° turns out to be negative (Δ*G*° < 0) when the temperature rises to 320 K. This indicates that the adsorption is non-spontaneous at lower temperatures, which turns to be spontaneous with increasing temperatures. The increase of temperatures facilitates desorption of water molecules. The more water molecules desorbed from the surface of M-PMs and PMs imply the MG molecules can be more effectively adsorbed onto the surface holes of M-PMs and PMs. It is thus more favorable for the adsorption of MG molecules with the bigger number of the water molecules desorbed. As a result, ΔG° shows a decreasing trend with increasing temperatures.

For the isothermal adsorption (see Figure 6c and Table 4), the adsorption of MG onto M-PMs is better applied to the Langmuir equation (as compared to the Freundlich equation), with the linear coefficient R^2^ located in the range of 0.85–0.95. Considering that the Langmuir equation assumes the mono-layer adsorption, the interactions between adsorbate molecules can be neglected. This thus reveals that the present M-PMs exhibit the mono-layer adsorption toward MG, thus satisfying the feature of chemical adsorption, in good agreement with the results as obtained by the kinetics and thermodynamics. Also note that the mono-layer adsorption amount, *q*_m_, and adsorption equilibrium constant, *k*_1_, show an increasing tendency with the increase of temperature, revealing that the increase of temperature facilitates the adsorption of MG onto M-PMs, in good consistence with thermodynamic results.

### 3.3. Evaluation of Adsorption Selectivity of M-PMs toward MG and the MISPE Column Applications

The structures of the five dye compounds, namely MG, RA, and BY, adopted for the specific recognition test are shown in Figure S3 of SM. RA has a structure similar to MG, both with the structure of three benzene rings, while BY presents a different structure from that of MG. Both M-PMs and PMs show stronger adsorption interactions with MG, followed by RA and then BY. The selectivity coefficient *k*’ is then used to further evaluate the adsorption selectivity of M-PMs and PMs. The *k*’ is a distribution coefficient ratio of template molecules to the competitive molecules during the adsorption, which can reflect the selectivity of M-PMs and PMs. M-PMs possess a higher *k*’ than PMs, both with the relative selectivity coefficient *K*_0_ bigger than 1 (Table 5). This therefore clarifies the higher selectivity of M-PMs toward MG as compared to PMs, as evidenced in Table 5.

**Figure 7:**
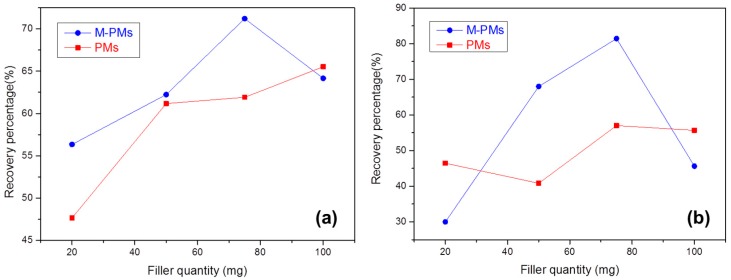
Recovering percentages of MG (**a**) and Leuco-MG (**b**), by using the Molecularly Imprinted Solid-Phase Extraction (MISPE) column filled with M-PMs or PMs.

For the MISPE applications, the percentage of MG and Leuco-MG, as recovered by the MISPE column filled with M-PMs, first increases with increasing the amount of M-PMs, and then lowers with an ongoing increase (Figure 7a,b). This optimal filling quantity of M-PMs is found to be 75 mg, leading to the recovering percentages of 71.2% and 81.4% for MG and Leuco-MG, respectively. Leuco-MG was also probed is by considering its structural similarity to MG and the ready transformation between MG and Leuco-MG under reductive and oxidative conditions. At an excessive low filling quantity of M-PMs, the resistance between the solution and MISPE column is too low, leading to a rather short time in penetrating the column and contacting with M-PMs. Consequently, such a short contacting time cannot enable a satisfactory adsorption of MG by M-PMs. On the contrary, when the filling amount of M-PMs is excessively large, e.g., 100 mg, the recovering percentage becomes lowered, which can be attributed to the fact that a long adsorption time makes it difficult to elute the adsorbed MG. On the other hand, it can be noted that M-PMs exhibit overwhelming recovery percentages in both MG and Leuco-MG cases as compared with PMs, revealing the significance of the molecular imprinting technology that can enhance the selective solid-phase extraction of MG and its Leuco derivative (Leuco-MG).

## 4. Conclusions

This paper has presented a systematic study on M-PMs prepared via Pickering emulsion polymerization combined with molecular imprinting with MG as the template. The structural features of the M-PMs have been well unraveled, along with its counterpart PMs without involving the MG template, by means of different kinds of characterization. Comparing to PMs that show an uneven porous surface (with roughness, a larger pore size, and higher specific surface area), the smaller M-PMs present a surface with regular pores of uniform pore size distribution, endowing M-PMs with an impressive selectivity to MG despite its lower adsorption capacity as a result of the lower specific surface area. It is more favorable for the static adsorption of MG from aqueous solutions under neutral and weakly acidic conditions. The overall endothermic adsorption of MG is primarily contributed by the endothermic water desorption process, with energy demand higher than the energy output as generated by the exothermic adsorption of MG. The chemical adsorption plays a dominant role in the adsorption of MG onto M-PMs, which enables a higher specificity of M-PMs for the adsorption toward MG relative to PMs. Detailed discussion and analysis of the adsorption kinetics and thermodynamics are also presented, which has been effectively used to explain the adsorption behaviors of M-PMs and PMs. The present work can pave the way for exploring various adsorbents with tunable structural features for detection and selective adsorption of a variety of organic pollutants. Light can also be shed on deep understanding of the adsorption interactions between various adsorbents and organic pollutants. The future work can be oriented to the optimization of the molecular imprinting conditions to further enhance the adsorption selectivity to MG or to other molecules of high concern nowadays.

## Figures and Tables

**Table 1:** Formulation used to synthesize molecularly imprinted polymer microspheres (M-PMs) and polymer microspheres (PMs) by Pickering emulsion polymerization.

Sample No.	Water Phase	Oil Phase					Result
	MG (mg)	EGDMA (mL)	MG (mg)	Leuco-MG (mg)	Styrene (mL)	Yield (%)	
1	0	1.73	0	0	0	93.1	Large diameter with uniformity
2	10	1.23	0	0	0.5	76.9	Fine flocculent particles, with lower color depth after styrene addition
3	0	1.73	10	0	0	91.9	Uniform and fine particles
4	10	1.23	0	0	0	83.3	Uniform and fine particles
5	0	1.23	0	5	0	81.1	Small diameter with uniformity
6	0	1.230	0	7.9	0	82.8	Small diameter with uniformity

Other conditions: in water phase, 20 mg nanosilica, 0.27 mL MAA, 0.5 mL NaOH solution (3 M), and 6 mL Tx-100 (0.3%); in oil phase, 200 µL toluene, and 20 mg initiator AIBN.

**Table 2:** Kinetic parameters for adsorption of MG with M-PMs and PMs.

Heading	*q_e_*, exp/(mg·g^−1^)	Pseudo-First-Order Kinetic Models			Pseudo-Second-Order Kinetic Models		
		*q*_1*e*_/(mg·g^−1^)	*k*_1_/(L·min^−1^)	*R*^2^	*q*_2*e*_/(mg·g^−1^)	*k*_2_/(L·min^−1^)	*R*^2^
PMs + MGM − PMs + MG	35.25	33.71	1.16 × 10^−3^	0.9416	38.52	3.89 × 10^−5^	0.9757
	30.47	30.25	7.14 × 10^−4^	0.9908	36.68	2.13 × 10^−5^	0.9942

**Table 3:** Thermodynamic functions calculated for adsorption of MG by M-PMs and PMs.

	Δ*S*°/(J/molK)	Δ*H*°/(KJ/molK)	*R*^2^	Δ*G*° 300 K/(KJ/mol)	Δ*G*° 310 K/(KJ/mol)	Δ*G*° 320 K/(KJ/mol)
PMs + MG	338.21	106.01	0.999	5.24	1.77	−1.51
M-PMs + MG	232.87	72.37	0.984	3.14	0.30	−1.49

**Table 4:** Fitting results of isothermal adsorption of MG by M-PMs.

T/K	Langmuir			Freundlich		
	*k*_l_/(L/mg)	*q*_m_/(mg/g)	*R*^2^	*k*_f_/(L/mg)	*n*	*R*^2^
298	0.13	3.14	0.975	2.13	1.78	0.885
308	1.48	3.15	0.950	2.00	5.84	0.831
318	0.12	4.71	0.962	1.59	2.07	0.751
328	1.58	6.02	0.916	3.64	4.32	0.761

**Table 5:** Partition and selectivity coefficients for PMs and M-PMs.

Comparing Composition	M-PMs			PMs			*K*_0_
	*C_e_* (mg/L)	*K*_d_ (L/g)	*k*′	*C_e_* (mg/L)	*K*_d_ (L/g)	*k*′	
MG	0.94	2.687		1.14	2.081		
RA	2.69	0.404	6.6	2.85	0.335	6.2	1.07
BY	2.72	0.236	11.3	3.25	0.190	10.9	1.03

Note: test conditions—at 35 °C, pH 7, initial dye concentration = 5 mg/L, dosage of microspheres = 0.03 g, and volume of the mixed solution = 25 mL.

## Supplementary Materials

The following are available online at www.mdpi.com/2073-4360/9/8/344/s1.
