# Supplementary material for: Combining Pickering Emulsion Polymerization with Molecular Imprinting to Prepare Polymer Microspheres for Selective Solid-Phase Extraction of Malachite Green
Source: Polymers (Basel). 2017 Aug 6;9(8):344. doi: 10.3390/polym9080344 (PMC6418669; doi:10.3390/polym9080344)
Supplement: Supplementary file 1 [file polymers-09-00344-s001.pdf]

polymers

Electronic Supporting Information

for

Combining Pickering emulsion polymerization with molecular imprinting to prepare polymer microspheres for selective solid-phase extraction of malachite green

Weixin Liang[1,2,†], Huawen Hu[1,†], Pengran Guo[2], Yanfang Ma[2,*], Peiying Li[1], Wenrou Zheng[1], Min Zhang[1,*]

[1] College of Materials Science and Energy Engineering, Foshan University, Foshan, Guangdong 528000, China
[2] Guangdong Provincial Public Laboratory of Analysis and Testing Technology, China National Analytical Center (Guangzhou), Guangzhou 510070, China
* Correspondence: myfedwin@126.com (Y.M.); zhangmin@gic.ac.cn (M.Z.)
† These authors contributed equally to this work.

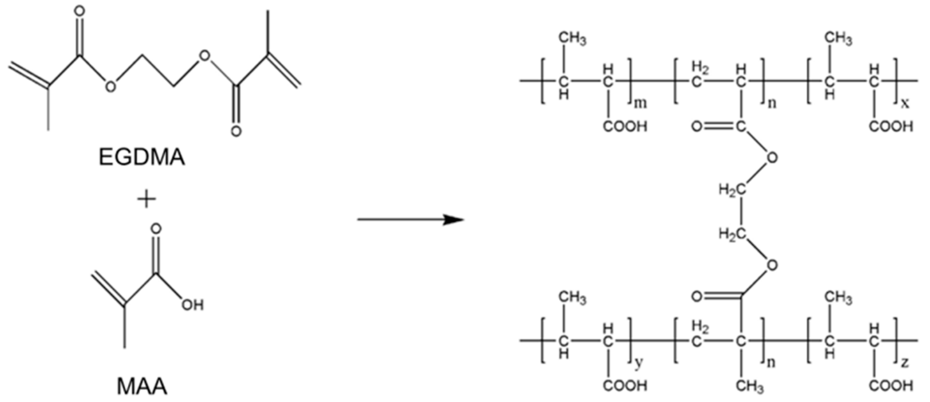

Figure S1 Molecular structures for showing the synthesis of polymer microspheres via free-radical polymerization and crosslinking reactions.

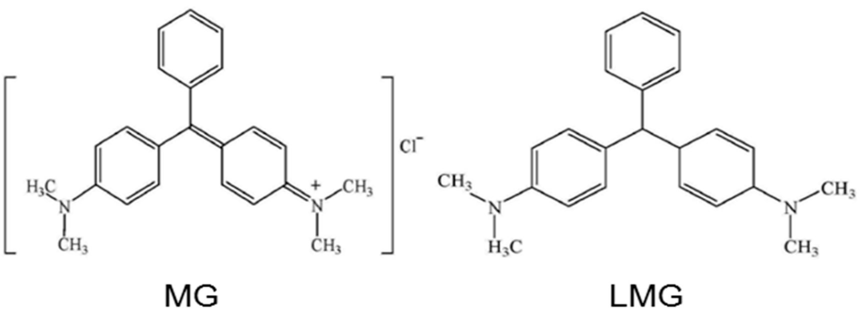

Figure S2 Molecular structures of MG and Leuco-MG (LMG).

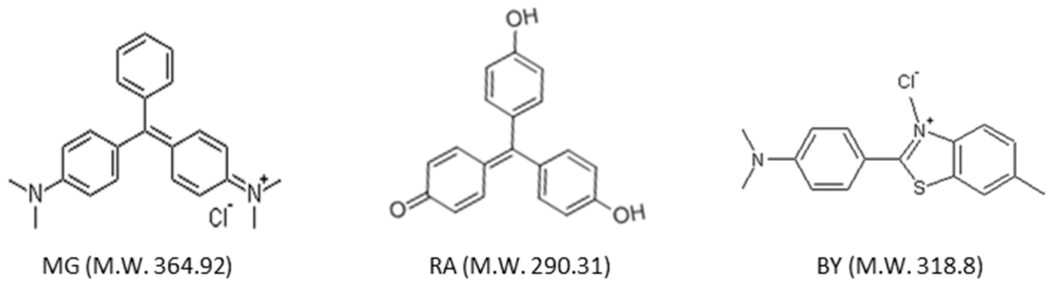

Figure S3 Molecular structures of MG, RA and BY, together with the corresponding molecular weight as given in the bottom row.

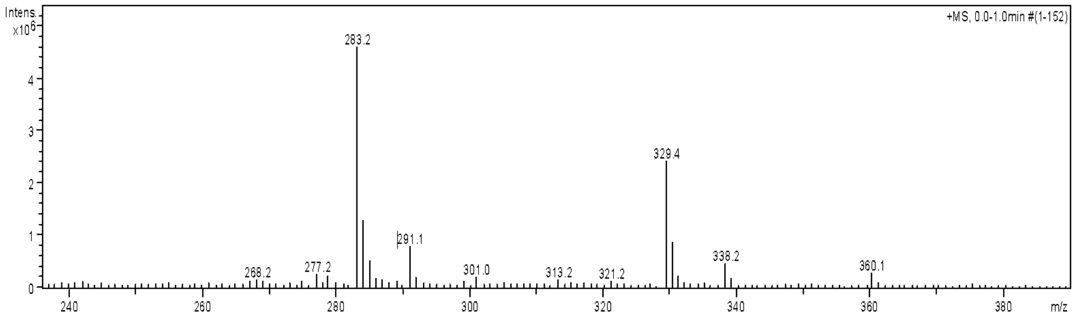

Figure S4 Mass spectra of MG, RA and BF.

In the ESI$^+$ ionization mode, the ion peaks at m/z291.1, m/z329.4 and m/z283.2 can be indexed to RA, MG (lack of a Cl^{-1} ion) and BY (lack of a Cl^{-1} ion), respectively.

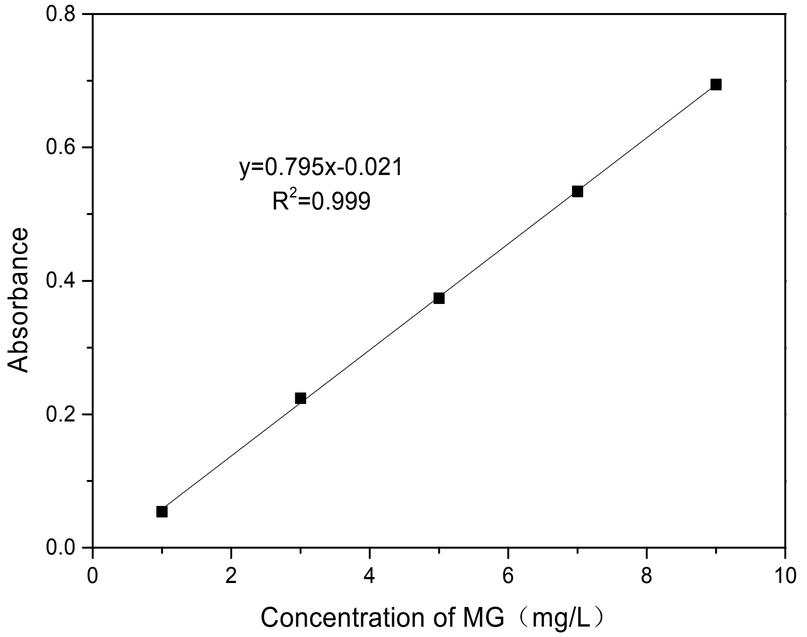

Figure S5 Standard curve of MG.

The MG concentration can be detected on a spectrophotometer. Specifically, MG standard solutions with concentrations of 1, 3, 5, 7, and 9 mg/L are measured, using DI water as the reference. The absorbance at 618 nm is monitored for obtaining the standard curve of MG.

Table S1 The number of microspheres, with a specific range of size, as counted for obtaining the particle diameter distribution histogram as shown in Figure 2(g).

Particle diameter (μm)	Number of M-PMs	Number of PMs
30~50	10	--
50~70	66	--
70~90	159	--
90~110	123	4
110~130	30	9
130~150	8	14
150~170	2	14
170~190	4	12
190~210	--	16
210~230	--	14
230~250	--	8

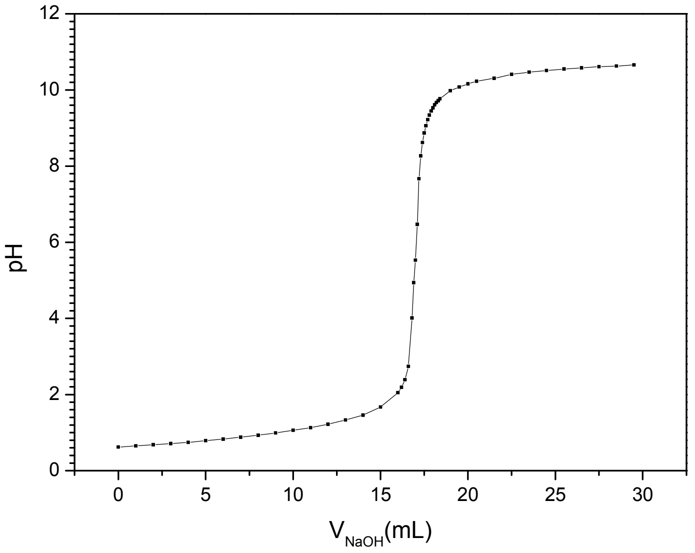

Figure S6 Determination of pKa of M-PMs by the titration method, which is estimated to be 5.85.
